# Macrophage phagocytosis alters the MRI signal of ferumoxytol-labeled mesenchymal stromal cells in cartilage defects

**DOI:** 10.1038/srep25897

**Published:** 2016-05-13

**Authors:** Hossein Nejadnik, Olga Lenkov, Florian Gassert, Deborah Fretwell, Isaac Lam, Heike E. Daldrup-Link

**Affiliations:** 1 Department of Radiology, Molecular Imaging Program at Stanford, Stanford University, Stanford, CA, USA

## Abstract

Human mesenchymal stem cells (hMSCs) are a promising tool for cartilage regeneration in arthritic joints. hMSC labeling with iron oxide nanoparticles enables non-invasive *in vivo* monitoring of transplanted cells in cartilage defects with MR imaging. Since graft failure leads to macrophage phagocytosis of apoptotic cells, we evaluated *in vitro* and *in vivo* whether nanoparticle-labeled hMSCs show distinct MR signal characteristics before and after phagocytosis by macrophages. We found that apoptotic nanoparticle-labeled hMSCs were phagocytosed by macrophages while viable nanoparticle-labeled hMSCs were not. Serial MRI scans of hMSC transplants in arthritic joints of recipient rats showed that the iron signal of apoptotic, nanoparticle-labeled hMSCs engulfed by macrophages disappeared faster compared to viable hMSCs. This corresponded to poor cartilage repair outcomes of the apoptotic hMSC transplants. Therefore, rapid decline of iron MRI signal at the transplant site can indicate cell death and predict incomplete defect repair weeks later. Currently, hMSC graft failure can be only diagnosed by lack of cartilage defect repair several months after cell transplantation. The described imaging signs can diagnose hMSC transplant failure more readily, which could enable timely re-interventions and avoid unnecessary follow up studies of lost transplants.

Human mesenchymal stem cells (hMSCs) are a promising tool for bone and cartilage regeneration^1^,^2^,^3^,^4^,^5^. hMSCs can be harvested from the patients’ own bone marrow, thus avoiding problems with immune rejection or ethical dilemmas associated with the use of human embryonic stem cells^6^,^7^,^8^. hMSCs can be easily expanded *ex vivo*, and clinical applications of hMSC transplants in cartilage defects have resulted in successful repair of full thickness cartilage defects^9^,^10^,^11^,^12^. However, apoptosis of hMSC transplants due to lack of growth factors, pro-inflammatory conditions or immune rejection is a common complication within the first weeks after transplantation. Currently, hMSC apoptosis and graft failure can be only diagnosed several months after cell transplantation, based on lack of cartilage defect repair^13^,^14^,^15^,^16^. An imaging technique that could diagnose hMSC death more readily would enable timely re-interventions and avoid unnecessary follow up studies of lost transplants.

Macrophages recognize dead cells based on specific surface characteristics, such as exposure of phosphatidylserine (PS) and calreticulin ligands on their surface membrane as well as loss of CD31 and CD47 surface markers^17^,^18^. Once they have recognized dead hMSCs, macrophages phagocytose them^19^,^20^. Capitalizing on this process, we recently found that pre-labeling macrophages with ultra-small superparamagnetic iron oxide nanoparticles can help to detect and track macrophage migration into dead stem cell implants, and thereby, indirectly diagnose cell death^21^. However, for many imaging applications, it would be advantageous to label the transplanted stem cells rather than macrophages. Labeling hMSC would allow us to address additional questions, such as correct deposition of hMSCs at the desired target site and evaluation of mechanical loss or migration of hMSC to other locations^22^,^23^,^24^.

Since macrophage phagocytosis may indicate graft failure, it is important to understand whether macrophage phagocytosis of apoptotic hMSC can be recognized by specific MR signal characteristics. To our knowledge, the influence of macrophage phagocytosis on the MR signal of iron oxide-labeled hMSCs in cartilage defects has not been evaluated. Thus, the purpose of our study was to evaluate if iron oxide-labeled hMSCs demonstrate different MR signal characteristics before and after phagocytosis by macrophages.

## Results

### Macrophages phagocytose iron labeled apoptotic stem cells and not iron labeled viable stem cells

**Figure 1:**
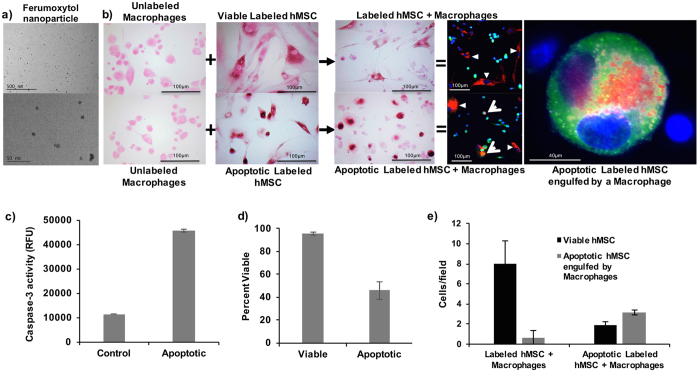
Macrophages phagocytose apoptotic but not viable stem cells. (**a**) TEM images of ferumoxytol nanoparticles. (**b**) DAB-Prussian blue stains of unlabeled macrophages, viable ferumoxytol-labeled hMSCs, apoptotic ferumoxytol-labeled hMSCs, viable ferumoxytol-labeled hMSCs co-incubated with macrophages, and apoptotic ferumoxytol-labeled hMSCs co-incubated with macrophages. DAB-Prussian blue positive iron is noted as brown staining in ferumoxytol labeled hMSCs and macrophages co-incubated with the apoptotic hMSCs. Fluorescence microscopy of Rhodamine-ferumoxytol labeled hMSCs, co-incubated with anti CD68 conjugated Alexa fluor 488 antibody labeled macrophages shows red-fluorescent ferumoxytol in the cytoplasm of green macrophages in samples with apoptotic hMSC, but not viable hMSC. DAPI was used as the nucleus marker. (**c**) The caspase assay shows increased expression of caspase-3 in apoptotic hMSC samples compared to viable hMSC samples. Data are displayed as means and standard deviation of three samples in each group. (**d**) The Trypan blue exclusion test confirms a higher percentage of dead cells in apoptotic compared to viable hMSC samples. Data are displayed as means and standard error of three samples in each group. (**e**) Apoptotic hMSC engulfed by macrophages were counted on confocal microscopy images as the number of cells with green, red and blue color. A higher quantity of triple color positive apoptotic hMSCs engulfed by macrophages was noted in samples with apoptotic hMSCs compared to samples with viable hMSC.

DAB-Prussian blue stains counterstained with Pararosaniline solution, showed positive iron staining of hMSCs after incubation with ferumoxytol (Fig. 1a), indicating nanoparticle uptake (Fig. 1b). Unlabeled control cells showed negative staining (Fig. 1b). Mitomycin C treated MSC showed a three-fold increase of caspase-3 activity (Fig. 1c) and 54% cell death on Trypan Blue stains (Fig. 1d). Apoptotic ferumoxytol-labeled hMSC co-incubated with macrophages showed positive iron stains of macrophages, suggesting hMSC phagocytosis. This was confirmed by confocal microscopy: CD-68 stained activated macrophages engulfed Rhodamine-ferumoxytol labeled apoptotic hMSC, but not Rhodamine-ferumoxytol labeled viable hMSCs (Fig. 1b). The number of viable hMSCs in the group of viable, iron labeled hMSC co-incubated with macrophages was four times higher than in the group of apoptotic, iron labeled hMSC co-incubated with macrophages (p = 0.0037). Accordingly, the amount of apoptotic hMSC engulfed by macrophages was three times lower (p = 0.0026) (Fig. 1e).

### Iron labeled viable MSC and iron labeled apoptotic MSC in macrophages cannot be distinguished *in vitro*

**Figure 2:**
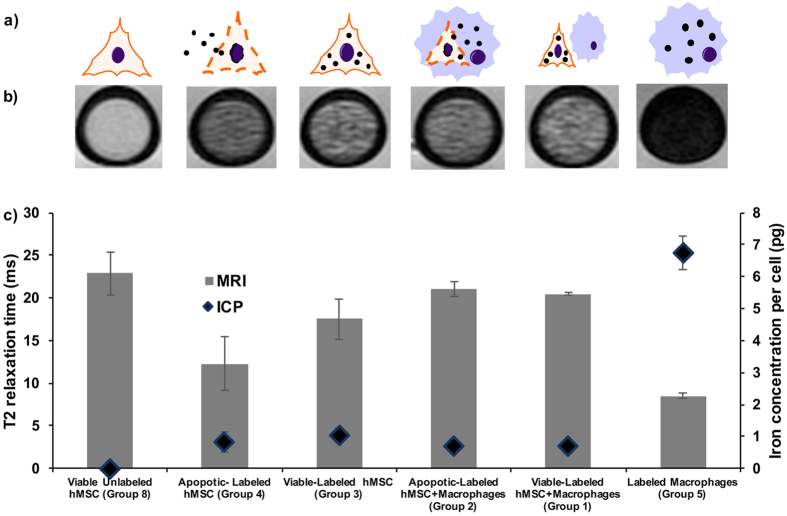
Iron oxide nanoparticle labeled viable MSC and iron oxide nanoparticle labeled apoptotic MSC in macrophages show no difference in MRI signal. (**a**) Graphic showing different experimental groups with different distributions and compartmentalizations of iron oxide nanoparticles (black dots) in hMSCs (triangular cell symbol), and macrophages (blue round cell symbol). (**b**) Axial T2 weighted (TE/TR = 30 ms/3000 ms) images of viable unlabeled hMSCs, viable/apoptotic labeled hMSCs, viable/apoptotic labeled hMSCs co-incubated with macrophages, and labeled macrophages in Ficoll in NMR test tubes. (**c**) Corresponding T2-relaxation times (grey bars) and iron content, quantified by ICP-OES (blue diamonds). Data are displayed as means and standard error of triplicate samples in each group.

Iron labeled hMSCs and iron labeled macrophages (groups 1–5) showed a decreased T2 signal compared to unlabeled controls (groups 6–10). T2-relaxation times of iron labeled cells were significantly shorter compared to unlabeled controls (p < 0.0001) and the corresponding intracellular iron content, as determined with ICP-OES, was significantly higher for the labeled hMSCs (group 1–5; Fig. 2a) compared to unlabeled hMSCs (group 6–9, p = 0.0074). Macrophages showed a higher phagocytic activity (group 5, 7.2pg per cell) compared to hMSCs (group 3, 1.2pg per cell) with a more hypointense MR signal (Fig. 2b), significantly higher cellular iron uptake (p = 0.0041) and significantly shorter T2-relaxation times (p = 0.0001) (Fig. 2c).

Iron labeled apoptotic hMSCs showed a more hypointense MR signal (Fig. 2b) and significantly shorter T2 relaxation times compared to iron labeled viable hMSCs, presumably due to release of iron oxides from apoptotic cells (p = 0.0141; Fig. 2c). We showed previously that free iron oxides show stronger T2 relaxation times compared to intracellular iron oxides^25^. Our current data show that there is no difference in MRI signal of iron oxide nanoparticles in hMSC and iron oxide nanoparticles in hMSC and macrophages: After co-incubation with macrophages, there was no significant difference in T2 values between viable iron labeled hMSCs besides macrophages and apoptotic iron labeled hMSCs in macrophages (p > 0.05). The intracellular iron content, as determined with ICP-OES, was not significantly different in cell samples of viable iron labeled hMSCs besides macrophages and apoptotic iron labeled hMSCs in macrophages (p > 0.05). The iron oxides were intracellularly compartmentalized in both cases (Fig. 1b).

### Iron labeled viable MSC and iron labeled apoptotic MSC in macrophages show different MRI signal kinetics *in vivo*

**Figure 3:**
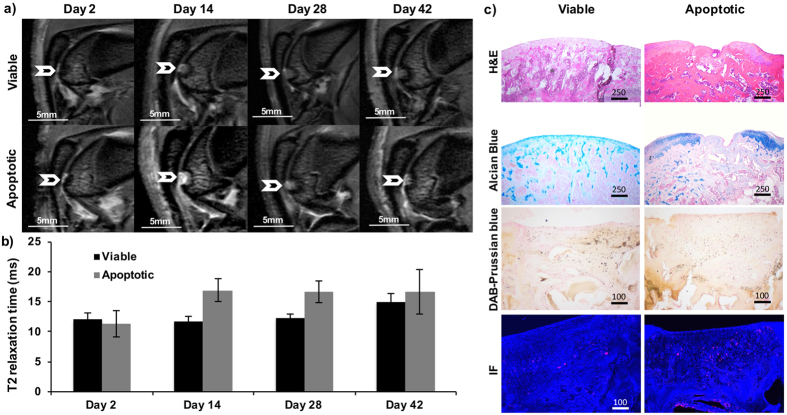
Iron labeled viable MSC transplants and iron labeled apoptotic MSC transplants in cartilage defects show different MRI signal kinetics over time. (**a**) Sagittal T2 weighted (TE/TR = 25 ms/3000 ms) MR images at different time points after implantation of viable (upper row) and apoptotic (lower row) hMSC in osteochondral defects of the distal femur of rat knee joints (arrow). (**b**) Corresponding T2 relaxation times of hMSC transplants, displayed as mean and standard error of five animals each with viable or apoptotic hMSC. T2 relaxation times of viable and apoptotic hMSC were significantly different on day 14 and 18 (p < 0.05). (**c**) Corresponding histopathology at day 42: Viable hMSC demonstrated successful engraftment in osteochondral defects, and successful defect repair, confirmed by smooth joint surface on H&E stains and chondrogenic matrix production on positive Alcian Blue stains. By comparison, apoptotic hMSC disappeared from osteochondral defects, as shown by unsuccessful defect repair, persistent defect on H&E stains and lack of chondrogenic matrix production on Alcian blue stains. DAB-Prussian blue stains show a higher amount of iron oxide nanoparticles in viable hMSC implants and immunofluorescence stains (IF) against CD68 positive macrophages (red) show a higher amount of macrophage invasion in apoptotic hMSC implants.

All ferumoxytol labeled hMSC transplants demonstrated a marked, negative signal on T2-weighted MR images directly after their transplantation into osteochondral defects of rat knee joints (Fig. 3a). However, MR signal kinetics of viable labeled transplants were different from apoptotic labeled transplants over time (Fig. 3a). Viable labeled transplants demonstrated a slowly increasing T2 signal over time, apparently a function of slow metabolism of the iron label (Fig. 3a). By comparison, the T2 signal of apoptotic labeled hMSCs increase faster (Fig. 3a). Corresponding T2 relaxation times of ferumoxytol labeled viable and apoptotic transplants were not significantly different on day 2 (p > 0.05), but did show significant differences on day 14 (p = 0.0118) and 28 (p = 0.0060) (Fig. 3b). On day 42, the T2 relaxation time of viable transplants becomes longer, suggesting dilution of ferumoxytol.

The differences in MRI signal at day 14 and 28 corresponded to different engraftment outcomes: Viable hMSC demonstrated successful engraftment in osteochondral defects, and complete defect repair, confirmed by a smooth joint surface on H&E stains and chondrogenic matrix production on positive Alcian blue stains (Fig. 3c). By comparison, apoptotic hMSC resulted in unsuccessful defect repair, as shown by persistent defect on H&E stains and lack of chondrogenic matrix production on Alcian blue stains at week 6 after the transplant. DAB Prussian-blue histology evaluation showed that iron oxide nanoparticles in apoptotic hMSC transplants disappeared faster from osteochondral defects compared to viable hMSC transplants, as shown by loss of brown-stained iron oxides in failed transplants in (Fig. 3c). At day 42, DAB Prussian blue stains showed less iron in the osteochondral defect containing apoptotic hMSC compared to the osteochondral defect with viable hMSC. CD68 immunofluorescence stains showed a higher quantity of macrophages in apoptotic transplants compared to viable transplants (Fig. 3c).

**Figure 4:**
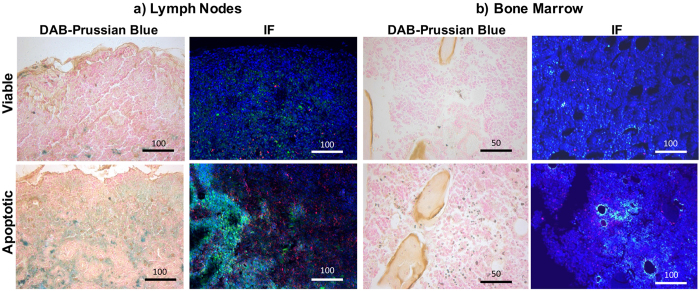
Migration of iron-loaded macrophages into surrounding bone marrow and regional lymph nodes. DAB-Prussian blue and CD68 immunofluorescence stains of (**a**) lymph nodes and (**b**) bone marrow of mice after transplantation of viable or apoptotic hMSC transplants. The higher amount of brown colored cells in the DAB-Prussian blue stains in apoptotic transplants suggests macrophages migration into both bone marrow and lymph nodes. Accordingly, immunofluorescence stains show a higher amount of ferumoxytol (green) and macrophages (red) in the bone marrow and popliteal lymph nodes of knee joints with apoptotic transplants.

To determine whether the faster disappearance of the iron label in apoptotic cell transplants was due to migration of iron-loaded macrophages out of the defect, we evaluated the adjacent bone marrow and regional lymph nodes for the presence of iron loaded macrophages by using Prussian blue and CD68 Immunofluorescence stains. We indeed found a higher amount of iron in both, bone marrow and lymph nodes of mice with apoptotic hMSC transplants compared to those with viable hMSCs, which can be explained by migration of macrophages after phagocytosis of iron-labeled apoptotic hMSCs (Fig. 4).

## Discussion

Our data showed that a rapidly declining T2-signal of iron-labeled stem cells in osteochondral defects indicates cell death and can predict incomplete defect repair weeks later. Currently, hMSC graft failure can be only diagnosed by lack of cartilage defect repair several months after cell transplantation. The described MR imaging signs can diagnose hMSC death more readily, which could enable timely re-interventions and avoid unnecessary follow up studies of lost transplants.

Our study is the first to investigate the effect of macrophages on the long-term MRI signal of iron oxide nanoparticle labeled viable and apoptotic stem cells. Previous investigators have addressed the critical question whether MRI is capable of distinguishing living iron labeled stem cells from dead iron labeled stem cells^26^,^27^,^28^,^29^. The results are conflicting and the question remains unsettled. Differences in research findings are likely attributable to differences in iron oxide nanoparticles, stem cell types, and tissue transplant sites.

There is some evidence from *in vitro* and *in vivo* studies, that longitudinal MRI studies reveal different MR signal characteristics of iron oxide labeled viable and dead cells^26^,^27^,^28^,^29^. Nedopil *et al.* and Hortelano *et al.* both described a decreased T2 signal of iron-labeled apoptotic MSCs and macrophages (RAW 264.7 cells) compared to viable controls. They hypothesized that the observed signal changes were attributable to physiochemical changes during cell degradation, which initially lead to clustering and subsequently release of iron oxide nanoparticles, and enabled increased interaction of nanoparticles with water protons^26^,^27^. *In vitro*, we have previously found that lysed cells release the iron oxide label (ferucarbotran) and the released nanoparticles provided a stronger T2 signal effect compared to intra-cellular compartmentalized iron oxides in MSCs^28^. Likewise, Kuhlpeter *et al.* found higher R2-values for free iron than compartmentalized iron (Resovist 45–60 nm in size) in a human lung carcinoma (CCL-185) cell line^29^.

While we found a faster decline in T2 enhancement of ferumoxytol labeled dead hMSCs compared to viable hMSCs in arthritic joints, other investigators reported a prolonged T2 signal of dead compared to viable ferumoxides-labeled stem cells in other organs like: (a) Cardiomyoblasts^30^,^31^,^32^: authors found a persistent MRI signal of iron-labeled myoblasts up to 3 months after implantation and concluded that MR imaging was inadequate for monitoring the viability of iron labeled myoblasts^33^, (b) The brain: authors have found that iron oxide labeled neural stem cells which underwent immune rejection produced a persistent hypointense MR signal after transplantation. Whereas the iron label was cleared more quickly from engrafting cells^34^,^35^. Dilution of the iron label due to cell division is perhaps more likely an issue in MR tracking of neural stem cells since it is essential in neuronal cell regeneration but not MSC-mediated cartilage regeneration. MSC in cartilage defects differentiate but usually do not proliferate; and (c) Skeletal muscle: Guenoun and colleagues reported a declining R2 and R2* signal of viable ferumoxides-labeled MSC and persistent R2 and R2* signal of nonviable ferumoxides-labeled MSC over 15 days after implantation into experimental mice^36^. The rapid metabolism of the iron label in this mouse model apparently indicated tumor formation in this mouse model. This is an important differential diagnosis to be considered at any transplants site, which could be further confirmed by signs of local stem cell proliferation and formation of a soft tissue mass.

Those differences in findings, however, may be due to differences in iron metabolism and macrophage behaviors in different organs. Our study showed in accordance with the above-described studies that the iron oxide signal on MRI images could persist beyond the time of death of labeled cells. However, we found different MR signal kinetic of iron oxide labeled viable and apoptotic MSC, which can be explained by two factors: (1) None of the above studies compared the MR signal characteristics of iron labeled dead cells with viable cells that successfully engrafted. One might argue that previous controls of presumed viable, but “mismatched” transplants in immune competent animals underwent apoptosis as well. Our studies included controls of viable transplants which successfully engrafted and repaired tissue defects, as proven by histology. (2) We investigated a unique environment of osteochondral defects, which is directly connected to an abundant source of macrophages (the bone marrow). This may have led to higher degree of macrophage influx into the transplant as compared to other tissue sites.

In accordance with our observations, Evgenov *et al.* found that iron oxide-labeled islet cells in the liver could be detected as dark, hypointense foci on T2* weighted MR images^37^. The MRI signal of the mismatched islet transplants in immunocompetent mice disappeared faster compared to islet transplants in immunodeficient mice, presumably due to faster clearance by macrophages.

In accordance with our earlier investigations^38^, we did not detect a difference in MRI signal of viable and nonviable MSCs in cartilage defects within the first few days after MSC transplantation. However, our current studies showed that MRI signal kinetics after two weeks were different between viable and apoptotic MSCs. The observed disappearance of the iron signal in apoptotic MSCs could be due to a more rapid metabolism of iron in macrophages (compared to iron in stem cells) or due to migration of ferumoxytol-hMSC-loaded macrophages from the transplant site to other sites. Our findings of iron loaded macrophages in bone marrow and lymph nodes support the latter hypothesis. This is further supported by previous reports of macrophage migration from sites of tissue injury to lymph nodes: Several studies investigated the fate of macrophages after phagocytosis of dead cells or other debris at inflamed sites and found that macrophages did not undergo local apoptosis but rather migrated to draining lymph nodes^39^,^40^,^41^,^42^. Accordingly, Cao *et al.* found that most macrophages migrated to local lymph nodes after they cleared debris in inflamed peritoneum, while only the minority of macrophages underwent local apoptosis^41^. Likewise, Lan *et al.* found that macrophages in experimental glomerulonephritis migrated to the marginal sinus of lymph nodes^42^ and Shakhbazau *et al.* reported that macrophages migrated from a spinal cord injury to local lymph nodes^43^.

Ferumoxytol (AMAG Pharmaceuticals) is a new iron oxide nanoparticle that recently approved by FDA as an iron supplement in patients with chronic kidney disease. Ferumoxytol is composed of an iron oxide core and a carboxymethyldextran coating (a low molecular weight semi-synthetic carbohydrate)^44^ with a hydrodynamic diameter of 30 nm. The carboxylated polymer shell of Ferumoxytol facilitates bio-functionalization of the Ferumoxytol coating surface^45^. The observed changes in MRI signal in our study are due to differences in iron oxide nanoparticle compartmentalization and metabolism. It has been previously described that nanoparticle size is an additional factor that affects the intensity and proportion of T1- and T2 signal effects of iron oxide nanoparticles on MR images^46^,^47^. Within the range of clinically applicable iron oxide nanoparticles with hydrodynamic diameters of 20 nm to about 150 nm^48^,^49^,^50^, the r2 relaxivity increases and the r1 relaxivity decreases with increasing nanoparticle size. Recently, larger superparamagnetic nanoparticles have been discontinued and are not available any more for clinical purposes. To date, ferumoxytol (Feraheme) and ferumoxtran-10 (Sinerem) are the only nanoparticle compounds available for clinical applications. Both agents have similar biochemical properties and similar relaxivities, and would therefore be expected to show similar results with regards to MRI signal effects of labeled cells and macrophage phagocytosis.

In conclusion, data showed that a rapid decline in T2-signal of iron-labeled stem cells in arthritic joints can be used as a predictive biomarker of unfavorable outcomes and lack of cartilage repair many weeks later. The described imaging signs can diagnose hMSC death more readily, which could be used to inform the development of more successful cellular therapies and ultimately, improved joint regeneration outcomes.

## Materials and Methods

### Do Iron labeled viable MSC and iron labeled apoptotic MSC in macrophages show different MRI signals *in vitro*?

#### Cell lines

The THP-1 cell line (Tamm-Horsfall protein-1 cell line, a human monocytic leukemia cell line; Sigma Aldrich, Saint Louis, MO, USA) has been well characterized and thoroughly established as a model for human monocytes and human macrophage-like cells^51^,^52^. THP-1 cells were grown on pre-treated polystyrene culture flasks at 37 °C and 5% CO_2_ in RPMI 1640 media (Sigma Aldrich, Saint Louis, MO, USA), supplemented with 10% fetal bovine serum (FBS) (Gibco, Grand Island, NY, USA), 100 units per mL of Penicillin and 100 mg per mL of Streptomycin (Gibco, Grand Island, NY, USA). THP-1 cells were activated into phagocytic macrophage-like cells using 1 μL Phorbol 12-Myristate 13-Acetate (PMA) (Fisher, Pittsburgh, PA, USA) per 10 mL of media according to previously established methods^51^,^53^,^54^. Briefly, THP-1 cells were activated with 160 nmol/L PMA for 72 hours to exhibit phagocytic behavior^55^.

hMSCs (Lonza, Walkersville, MD, USA, Cat No. PT-2501)^56^,^57^,^58^,^59^ were grown at 37 °C and 5% CO_2_ in complete culture medium consisting of high-glucose Dulbecco’s Modified Eagle Medium (DMEM) (Gibco, Grand Island, NY, USA, Cat. No. 11965118) supplemented with 10% FBS and 100 units per mL of Penicillin and 100 mg per mL of Streptomycin. Media was changed every 2–3 days and cells were used up to passage 10. When cells reached 80% confluency, they were labeled with ferumoxytol nanoparticles (Feraheme™, AMAG Pharmaceuticals; 500 μg Fe/mL media) using the transfection reagent Lipofectin^®^ (Life Technologies) as previously described^60^,^61^. After 24 hours of incubation with the labeling media, cells were washed three times with PBS. A subset of hMSCs was incubated with 0.06 mg Mitomycin-C per ml media for apoptosis induction via the p53-mediated pathway^62^,^63^. This method has previously revealed a significant increase of caspase activity of Mitomycin-C treated hMSCs, as determined with the Caspase 3/7 kit (SensoLyte AMC Caspase 3/7 assay kit, Anaspec, San Diego, CA, USA)^26^. We used the same Caspase 3/7 kit to confirm hMSC apoptosis in our Mitomycin-treated cell samples^64^. In addition, a Trypan Blue test was used to determine the percentage of viable cells in the samples.

#### Experimental groups

Cells were randomly divided into the following groups:

*Group 1:* viable, iron oxide nanoparticle-labeled hMSCs co-incubated with human monocytes

*Group 2:* apoptotic, iron oxide nanoparticle-labeled hMSCs co-incubated with human monocytes

*Group 3, 4:* Control groups comprised of iron oxide nanoparticle-labeled viable and apoptotic hMSCs

*Group 5:* Control group of iron oxide nanoparticle-labeled monocytes

*Group 6–10:* Unlabeled samples corresponding to groups 1–5

Triplicate cell samples in all groups underwent DAB-Prussian blue staining, inductively coupled plasma atomic emission spectrometry (ICP-OES), immunohistochemistry, and MR imaging.

#### Histopathology and ICP

Iron uptake of cell samples from all groups was evaluated qualitatively by DAB-Prussian blue staining with the Accustain Iron Stain kit (Sigma-Aldrich, St. Louis, MO, USA) and a Pararosaniline solution counterstain. Cellular iron uptake was quantified by inductively coupled plasma atomic emission spectrometry (ICP-OES) (Perkin-Elmer, Waltham, MA, USA). In order to detect hMSC phagocytosis by macrophages, the hMSCs were labeled with Rhodamine-conjugated ferumoxytol, using the above-described labeling approach^65^ and divided into group 1–5 as described above. Macrophages were stained with FITC 488-conjugated anti-CD68 monoclonal antibody (Abcam), and cell nuclei in the samples were stained with 4’, 6-Diamidin-2-phenylindol (DAPI; Invitrogen). The amount of viable hMSCs and apoptotic hMSCs engulfed by macrophages was counted in quadruplicate samples of labeled hMSCs co-incubated with macrophages and apoptotic labeled hMSCs co-incubated with macrophages in high power field (400× magnification) after staining with the above-described labeling approach.

#### MR Imaging

Triplicate samples of 600,000 cells per group 1–10 were suspended in 30 μl of isotonic (1.07 g/mL) Ficoll solution, transferred to 3 mm diameter NMR tubes and scanned on a 7T MR scanner (hybrid Varian magnet, GE Medical Systems interface, Milwaukee, WI, USA) using a custom-made partial birdcage coil. The test tubes were placed in a PBS-containing plastic container to avoid susceptibility artifacts from surrounding air. For measurements of T2 relaxation times, axial spin echo (SE) sequences were obtained with a repetition time (TR) of 3000 ms and multiple echo times (TE) of 80, 60, 40, and 20 ms. The images were acquired with a field of view (FOV) of 25 × 25 mm, a matrix of 256 × 256, a slice thickness of 0.5 mm and two acquisitions. MR dicom data were processed by the Cine tool software program (CINE Tool, GE Healthcare Technologies, Baltimore, MD, USA), which calculated T2-relaxation time maps. T2-relaxation times of every cell samples were derived by region of interest (ROI) measurements of the test samples on these maps.

### Do Iron labeled viable MSC and iron labeled apoptotic MSC in macrophages show different MRI signals *in vivo*?

#### Stem cell transplantation into cartilage defects

The study was approved by Administrative Panel on Laboratory Animal Care (APLAC) of Stanford University and all the methods were carried out in accordance with the approved guidelines. In five athymic female Harlan rats, osteochondral defects were created in the distal femur of both knee joints under inhalation anesthesia. A medial patellar skin incision was made, the patella was dislocated laterally and a circular osteochondral defect (diameter: 2 mm, depth: 1.5 mm) was created in the distal femoral trochlear groove using a micro-drill (Saeyang, Daegu, Korea). In these defects, either 7.5 × 10^5^ ferumoxytol labeled viable hMSCs (right femur, n = 5), or ferumoxytol labeled Mitomycin-pretreated hMSCs (left femur, n = 5) were implanted, using an agarose scaffold (5 μl, Type VII, Sigma Aldrich, St Louis, MO, USA). We have previously shown, that Mitomycin-pretreated hMSC undergo apoptosis *in vivo* within 24 h after transplantation^21^,^66^. After allowing the agarose scaffold to solidify for 1–2 minutes, the patella was repositioned and skin incision was closed by Dermalon 6-0 monofilament sutures.

#### MR Imaging

The hMSC transplants were investigated with MR imaging 2 days as well as 2, 4, and 6 weeks after hMSC implantation. Rats were sacrificed 6 weeks after hMSC implantation for histologic correlations of imaging data.

MR imaging of all knee joints was performed with the same 7T MR scanner and RF coil used for *in vitro* studies. Animals were anesthetized with 2% isoflourane and placed supine on a custom-built trough with their knees centered in the RF coil. Sagittal MR images of the rat knee joints were obtained with a fast spin echo (FSE) sequence (TR 3000 ms, TE 25 ms, number of excitation (NEX): 12, FOV: 2.5 × 2.5 cm, Matrix: 256 × 256, Slice thickness: 0.5 mm and a multi-echo spin echo (SE) sequence (TR 4000 ms/TE 15, 30, 45, and 60 ms), using a field-of-view (FOV) of 25 × 25 mm, a matrix of 256 × 256, and a slice thickness of 0.5 mm. T2 relaxation times of each cell implant was measured via operator-defined ROIs.

#### Histopathology

After the last MRI scan, each animal were sacrificed, knee joints were resected and placed in Cal-Ex II (Fisher Scientific) for 4–5 days. Cal-Ex II is a mixture of formaldehyde and formic acid, which decalcifies the bones and fixes the tissue simultaneously. Decalcified specimen were dissected parasagittally, dehydrated through graded alcohol washes, embedded in paraffin, cut in 5 μm sections and stained with standard haematoxylin and eosin (H&E) to define the morphology of the implant and Alcian blue to confirm hyaline cartilage matrix production in the viable implants. DAB-Prussian blue stains and anti-CD68 stains were performed on deparaffinized sections similar to *in vitro* studies (see above). Anti-CD68 staining specific for ED-1 macrophages (Primary Antibody: Mouse anti-rat CD68, Abcam, Cambridge, MA, USA; Secondary Antibody: Alexa flour 647 goat anti mouse, Invitrogen, Eugene, OR, USA) was added to detect macrophages in the transplant and anti-Dextran, Clone DX1, FITC (Mouse Anti-Dextran, FITC-conjugated, Stem Cell Technology, Tokwila, WA, USA) to detect ferumoxytol labeled hMSC.

### Statistical Analysis

For each experimental group, quantitative data were expressed as mean data and standard error. Data of different *in vitro* and *in vivo* groups were compared using Student’s t-test. For all analyses, a p-value of less than 0.05 was considered to indicate significant differences.

## Additional Information

**How to cite this article**: Nejadnik, H. *et al.* Macrophage phagocytosis alters the MRI signal of ferumoxytol-labeled mesenchymal stromal cells in cartilage defects. *Sci. Rep.* **6**, 25897; doi: 10.1038/srep25897 (2016).
